# A Novel Pseudopodial Component of the Dendritic Cell Anti-Fungal Response: The Fungipod

**DOI:** 10.1371/journal.ppat.1000760

**Published:** 2010-02-12

**Authors:** Aaron K. Neumann, Ken Jacobson

**Affiliations:** Department of Cell & Developmental Biology, University of North Carolina at Chapel Hill, Chapel Hill, North Carolina, United States of America; University of Birmingham, United Kingdom

## Abstract

Fungal pathologies are seen in immunocompromised and healthy humans. C-type lectins expressed on immature dendritic cells (DC) recognize fungi. We report a novel dorsal pseudopodial protrusion, the “fungipod”, formed by DC after contact with yeast cell walls. These structures have a convoluted cell-proximal end and a smooth distal end. They persist for hours, exhibit noticeable growth and total 13.7±5.6 µm long and 1.8±0.67 µm wide at the contact. Fungipods contain clathrin and an actin core surrounded by a sheath of cortactin. The actin cytoskeleton, but not microtubules, is required for fungipod integrity and growth. An apparent rearward flow (225±55 nm/second) exists from the zymosan contact site into the distal fungipod. The phagocytic receptor Dectin-1 is not required for fungipod formation, but CD206 (Mannose Receptor) is the generative receptor for these protrusions. The human pathogen *Candida parapsilosis* induces DC fungipod formation strongly, but the response is species specific since the related fungal pathogens *Candida tropicalis* and *Candida albicans* induce very few and no fungipods, respectively. Our findings show that fungipods are dynamic actin-driven cellular structures involved in fungal recognition by DC. They may promote yeast particle phagocytosis by DC and are a specific response to large (i.e., 5 µm) particulate ligands. Our work also highlights the importance of this novel protrusive structure to innate immune recognition of medically significant *Candida* yeasts in a species specific fashion.

## Author Summary

Yeasts are normal microbial commensals of humans and a significant source of opportunistic infections, especially in immunocompromised individuals. We report a novel cellular protrusive structure, the fungipod, which participates in the host-microbe interaction between human immature dendritic cells (DC) and yeasts. The fungipod's structure is based on and propelled by a robust process of local actin cytoskeleton growth at the DC-yeast contact site, and this cytoskeletal remodeling results in a durable tubular structure over 10 µm long connecting the dorsal DC membrane and yeast. The fungal cell wall polysaccharides mannan and chitin trigger fungipod formation by stimulating the carbohydrate pattern recognition receptor CD206. Fungipods are part of a specific response to large particulate objects (i.e., yeast), and they may promote the human immature DC's relatively poor phagocytosis of yeast. The human fungal pathogen, *Candida parapsilosis*, induces a strong fungipod response from DC, and this response is highly species specific since the related pathogens *Candida albicans* and *Candida tropicalis* induce fungipods rarely. Our work highlights a novel cell biological element of fungal recognition by the innate immune system.

## Introduction

The innate immune response against fungal pathogens is effected by macrophages, neutrophils and dendritic cells (DC). DC may encounter opportunistic fungi, such as *Candida* species, in cutaneous and mucosal tissue as well as in disseminated infections associated with serious disease. The innate immune response relies on two classes of pattern recognition receptors, the Toll-like receptors (TLR) and C-type lectin receptors (CLR) that permit the apprehension of non-self ligands.

Infection by *Candida* species yeasts represents an important opportunistic infectious disease threat that must be constantly countered by the innate immune system. *Candida* is ubiquitous and exists as a normal commensal microbe on human mucosal and cutaneous surfaces. However, *Candida* is also an opportunistic human fungal pathogen often infecting intensive care, post-surgical and neutropenic patients. The immunocompromised population has grown in modern times with the spread of AIDS and more widespread use of immunosuppressive therapies in groups like solid organ transplant recipients. These patients are generally at risk for fungal infection. In the United States, *C. albicans* accounts for the majority (70–80%) of clinical fungal isolates; however, other species (*C. glabrata*, *C. tropicalis*, *C. parapsilosis* and *C. krusei*) are emerging fungal pathogens with *C. glabrata* and *C. tropicalis* accounting for 5–8% of clinical isolates [1],[2]. In Europe, Asia, and South America, the incidence of *C. parapsilosis* infection can exceed that of *C. albicans*, and *C. parapsilosis* is the most rapidly emerging *Candida* species since 1990 [3].

The fungal cell wall contains mannan, β-glucans, chitin and other carbohydrates recognizable as non-self epitopes [4],[5]. These cell wall carbohydrates are ligands for CLRs. Dectin-1, DC-SIGN and CD206 (Mannose Receptor) are transmembrane CLRs expressed on immature DC that contribute to recognition of yeasts [6]. Dectin-1 binds β-(1,3)-glucans and contains a cytoplasmic ITAM motif allowing Syk-dependent activation of phagocytosis and cytokine production. Plasma membrane microdomains of DC-SIGN in immature DC contribute to avid binding of high mannose carbohydrates such as mannan [7],[8]. CD206 binds terminal mannose, fucose and N-acetyl glucosamine residues conferring binding activity for mannan and chitin in the yeast cell wall [9],[10],[11]. CD206 participates in fungal acquisition, cytokine elaboration and phagocytosis of yeast [12],[13],[14].

Internalization is central to many aspects of CD206 biology. Indeed, the majority of CD206 is found within the endocytic system, and internalization of antigen via CD206 in DC results in MHC class II antigen presentation for T cell activation [15]. Furthermore, CD206 also has important homeostatic functions in endocytically removing hydrolases, tissue plasminogen activator and myeloperoxidase during inflammatory responses [9],[16],[17]. The cytoplasmic tail contains a dihydrophobic motif involved in endosomal sorting and a tyrosine-based FxNxxY internalization motif similar to that found in the cytoplasmic tail of LDL receptor [18]. The latter motif supports attachment of CD206 to clathrin lattices via AP-2 leading to internalization within clathrin-coated vesicles (CCV).

Dendritic actin network polymerization via Arp2/3 is a recurrent theme in host cell-microbe interactions. Arp2/3 complex binds to existing F-actin and upon activation nucleates new filament growth from this branch point [19],[20]. DC express the Arp2/3 activators, WASP and cortactin. Cortactin-mediated actin remodeling is co-opted by numerous microbial pathogens [21]. Cortactin stabilizes Arp2/3 branch points driving dynamic actin structures such as lammelipodial protrusions [22], actin pedestals [23], and actin comet tails [24]. Serine and tyrosine phosphorylation as well as physical recruitment variously determine the location and timing of cortactin activity [25],[26],[27],[28]. For example, dynamin recruits cortactin to clathrin-coated pits during vesicle scission [29],[30],[31] leading to actin polymerization around the nascent vesicle neck.

Activation of the actin remodeling machinery is a normal part of the professional phagocyte's response to microbes (although it is sometimes co-opted by pathogens). This machinery must be directed by pattern recognition receptors to recognize invading microbes. In this report we describe a novel actin-based protrusive structure formed by DC in response to ligation of CD206 by yeast cell walls.

## Results

### Morphology of Zymosan-Induced DC Fungipods

**Figure 1:**
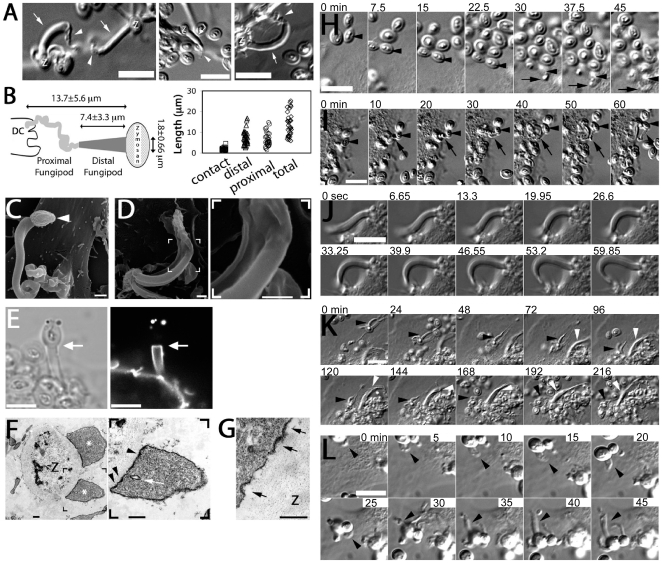
Morphology and formation of fungipods. (A) DIC images of representative fungipods on the dorsal surfaces of immature DC after 4 hours exposure to zymosan. “Z” denotes the location of the fungipod attached zymosan particle. Arrows and arrowheads designate distal and proximal fungipod regions, respectively. Bars = 10 µm. (B) Schematic of fungipod morphology and distribution of measured contact site widths as well as lengths of distal, proximal and total fungipod regions (N = 35). (C) SEM image of a typical fungipod with roughly cylindrical distal geometry (9500x). Arrowhead denotes the attached zymosan particle. Bar = 1 µm. (D) SEM image of a fungipod with ribbon-like distal geometry displaying longitudinal ridges. Right panel shows a higher magnification view of the flattened structure in the distal fungipod from the bracketed area of the left panel. Left panel, 7500x; Right panel, 25000x, Bar = 1 µm. (E) DiI-labeled membrane of a distal fungipod at the zymosan contact site (arrow) viewed as a medial confocal section of the distal fungipod. (left, DIC; right, confocal fluorescence). Bars = 5 µm. (F) A fungipod/zymosan contact site seen in thin section TEM imaging. The right panel displays the left panel bracketed region at higher magnification. Designations: “Z”, Zymosan; “*”, distal fungipod; arrow, example of vesicle inside distal fungipod; arrowheads, examples of pits/membrane densities at the contact site. Left panel, 2700x; Right panel, 6500x; Bars = 500 nm. (G) Thin section TEM of membrane invaginations at a zymosan contact site displaying studded juxtamembrane densities. 11000x, Bar = 500 nm. (H) DIC imaging of the initial attachment and nascence of a fungipod. Arrowheads and arrows designate a zymosan particle associated with fungipod generation and the nascent fungipod, respectively. Times indicate elapsed time of DC attachment for the indicated zymosan particle. Bar = 10 µm. (I) DIC imaging of the maturation of a fungipod. Arrowheads and arrows designate a relevant zymosan particle and the growing fungipod, respectively. Times indicate elapsed time since the advent of the fungipod. Bar = 10 µm. (J) DIC imaging of the growth of a mature fungipod. Bar = 10 µm. (K) DIC imaging of fungipods (arrowheads) associated with zymosan and DC for several hours. Bar = 10 µm. (L) DIC imaging of a fungipod (arrowhead) formed by a DC in response to live *S. cerevisiae*. Bar = 10 µm.

We found that human monocyte-derived DC generated peculiar dorsal pseudopodial structures after several hours of exposure to zymosan. We designated these protrusions “fungipods”. They were visible in DIC imaging, resembling long tethers connecting DC and zymosan, and were comprised of an apparently smooth, well-ordered distal region tapering into a convoluted cell-proximal region. Distal fungipods averaged 7.4±3.3 µm long (N = 35, range: 2.7–16.8 µm) and 1.8±0.67 µm wide (range: 0.92–4.6 µm) at the contact site. Their overall length was 13.7±5.6 µm (range: 5.9–25.3 µm) (Figure 1A, B). Most distal fungipods appeared roughly cylindrical in SEM (Figure 1C), but we occasionally observed ribbon-like fungipods with longitudinal ridges (Figure 1D). This variety suggested that the cross-sectional shape of the distal fungipod was determined by the geometry of the contact site on the zymosan particle. SEM imaging and DiI labeling of contact site membranes revealed a contact site structure limited to the footprint of the fungipod with no fungipod membrane extended outside of the fungipod contact site footprint visible in DIC (Figure 1E and data not shown). The fungipod plasma membrane was tightly apposed to zymosan, and electron dense juxtamembrane patches were observed by TEM in the fungipod plasma membrane present at the zymosan contact site (Figure 1F, G). Interestingly, these membrane densities were often associated with membrane pits and displayed knobbed or studded juxtamembrane densities. In Figure 1F, the zymosan is contacted by two fungipods, and the lower one is detailed to emphasize these juxtamembrane densities. Finally, we found that zymosan-treated human monocyte derived immature macrophages produce fungipods similar to those on DC (Figure S1A). Activation of human immature DC or macrophages with LPS significantly attenuates fungipod formation efficiency, although fungipods are not completely abolished on LPS-activated cells (Figure S1B).

Fungipodial structures required association of zymosan particles with the plasma membrane. Blocking with excess soluble mannan plus the β-glucan laminarin completely inhibits interaction of zymosan with CLR on DC [14], and this prevented binding and fungipod formation (data not shown). Time-lapse DIC imaging revealed that the initial fungipod extension was typically observable from ∼0.5–2 hours post-attachment and protrusions were only observed to form next to and bind to zymosan after prolonged association with DC (Figure 1H; Video S1). The fungipods in this example grew from a plane below the focus. We have not observed fungipods to form independent of surface associated zymosan. This demonstrates that the fungipodial structure is a response to a previously ligated particle and is not a pre-formed protrusion searching for a ligand.

Early zymosan-induced fungipodial extensions were often thin and lacked a well-ordered distal fungipod, but they matured into the previously described fungipods over the course of approximately 45–60 minutes (Figure 1I; Video S2). These mature fungipods exhibited steady growth and movement (Figure 1J). Zymosan was firmly bound by fungipods and consistently remained stably associated over hours (Figure 1K).

We confirmed that DC form fungipods after exposure to live cultures of budding *S. cerevisiae*. These protrusions exhibited a morphology identical to those observed for zymosan particles (Figure 1L).

**Figure 2:**
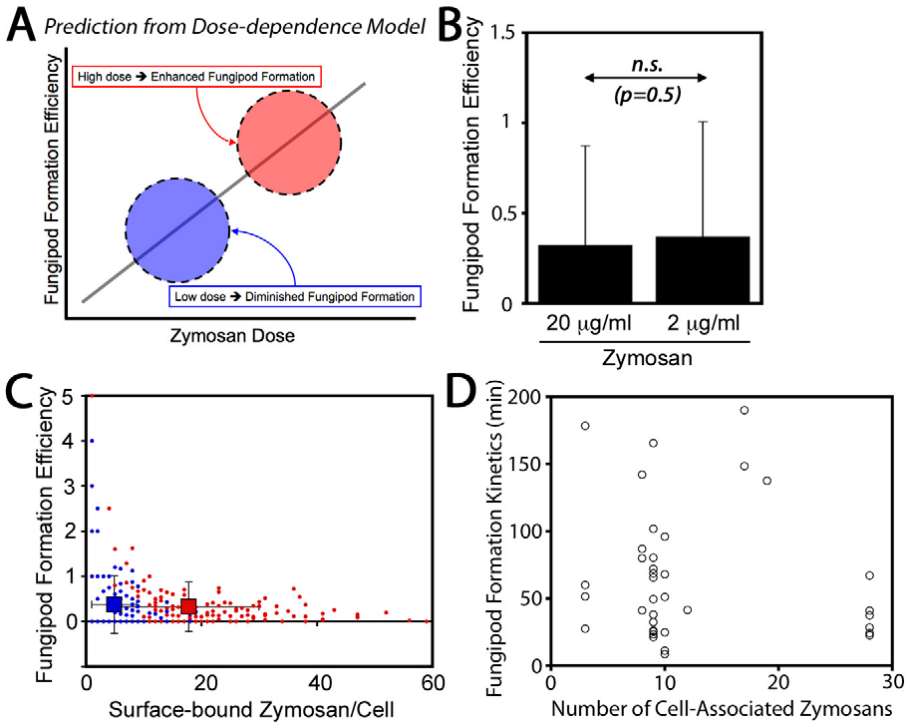
Zymosan dose-dependence of fungipods. (A) Dose-dependence of fungipods predicts higher fungipod formation efficiencies at higher degrees of DC stimulation by zymosan particles. (B) Fungipod formation efficiency in DC-zymosan cultures is independent of zymosan density applied to DC. (n.s., not significant by Student's t-test) (C) Individual DC show no positive correlation between degree of zymosan stimulation and fungipod formation efficiency. For panels B & C, N = 165 cells for 20 µg/ml zymosan and N = 145 for 2 µg/ml zymosan. (D) There is no apparent correlation between degree of DC stimulation by zymosan and the kinetics of fungipod formation (N = 39 individual fungipod formation events).

Finally, we also considered that fungipod formation might arise only in DC highly stimulated via the acquisition of many zymosan particles. In this dose-dependence model, fungipod formation efficiency would be positively correlated with zymosan dose such that high fungipod formation efficiencies would be predicted at high dose and vice versa (Figure 2A). To address this possibility we treated DC with our standard zymosan particle density (20 µg dry weight/ml) and a one log lower density (2 µg/ml). We found that the fungipod formation efficiency under these two regimes was statistically indistinguishable by Student's t-test (p = 0.5) (Figure 2B). Another prediction of the dose-dependence model is that within an experiment, cells that happen to bind larger numbers of zymosan particles will have higher fungipod formation efficiency. We plotted efficiency versus the number of surface-bound zymosan particles and found that, regardless of the zymosan density applied to the DC culture, individual DC with higher zymosan loads generally had low fungipod formation efficiencies (Figure 2C). We found similar trends if the number of internalized zymosan particles or the total number of cell-associated zymosan particles was plotted on the abscissa. We also found no correlation between the number of cell-associated zymosan particles and the kinetics of fungipod formation (time from binding to initial fungipod extension) (Figure 2D). Therefore, there is no positive correlation between degree of global zymosan stimulation and fungipod formation efficiency or fungipod formation kinetics in immature DC.

### Cytoskeletal Structure of Fungipods

**Figure 3:**
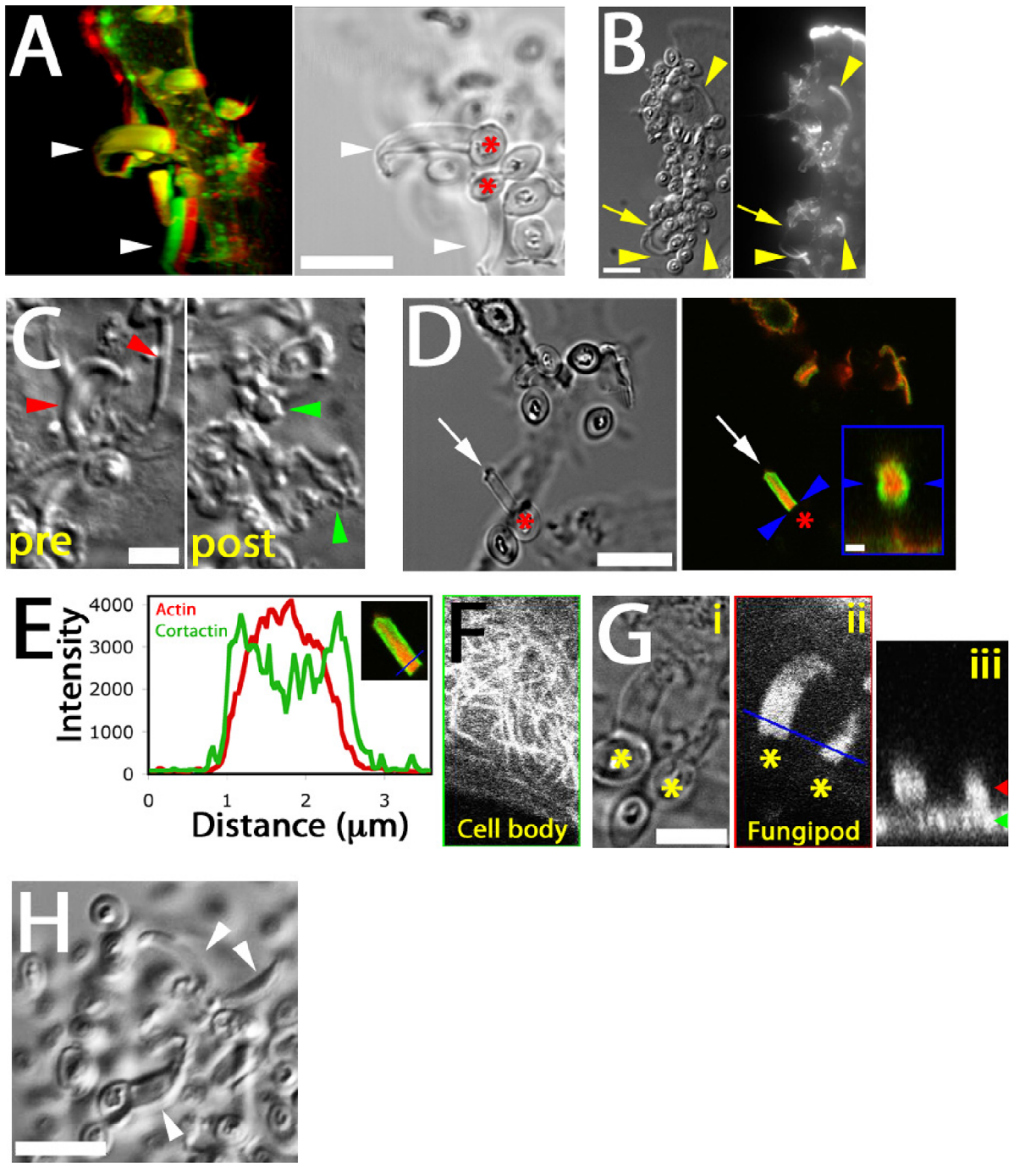
Cytoskeletal structure of fungipods. (A) Left panel: Red/green anaglyph of actin in DC fungipods (arrowheads) after 4 h exposure to zymosan. Non-fluorescent zymosan particles contacted the distal fungipod ends. Right panel: A single z-axis plane DIC image showing the position of zymosan particles (asterisk) with respect to fungipods (arrowhead). Bar = 10 µm. (B) Images of zymosan induced fungipods (arrowheads) in DIC (left panel) and actin-stained epifluorescence (right panel). The DIC image represents a single z-axis plane above the dorsal membrane, and actin is shown as a maximum intensity projection of all z-axis planes. Arrow indicates proximal fungipod. Bar = 10 µm. (C) DIC images of 4 hour zymosan-exposed DC fungipods before (“pre”) and after (“post”) treatment with cytochalasin D (15 minutes, 1 µm). Fungipods were well ordered before treatment (red arrowheads) and disordered after treatment (green arrowheads). Bar = 5 µm. (D) DIC (left panel) and confocal fluorescence imaging (right panel) of fungipods on DC after 4 hour zymosan exposure showing actin in red and cortactin in green at a fungipod-medial z-axis plane. Arrow and asterisk denotes fungipod and attached zymosan, respectively. Bar in left panel = 10 µm. Arrowheads in right panel designate the contact site and right panel inset is an orthogonal section through the contact site. Inset bar = 1 µm. (E) Linescan of actin (red) and cortactin (green) intensities (arbitrary units) in the distal fungipod. Location of line is shown in the inset image. (F) Confocal imaging of α-tubulin in the DC cell body showing microtubules. (G) (i) DIC image of fungipods at a medial z-axis plane with zymosan particles marked by asterisks. (ii) Diffuse α-tubulin staining in the distal fungipod. Blue line represents the region used for orthogonal section. (iii) Orthogonal section of previous panel showing fungipod diffuse tubulin in cross-section. Green and red arrowheads denote the z-axis positions of the cell body and fungipod images, respectively. Bar = 5 µm. (H) Fungipods (arrowheads) persist even after 1 hour exposure to nocodazol (10 µM). Bar = 10 µm.

Distal fungipods contained copious F-actin in a structure tapering away from the contact site (Figure 3A, B; Video S3). The convoluted proximal fungipods typically exhibited weaker F-actin signals (Figure 3B, arrow). Existing fungipods soon collapsed upon addition of the F-actin elongation blocker cytochalasin D leaving disordered membranous extensions (Figure 3C). These data demonstrate that a dense actin structure is necessary to maintain the distal fungipod and suggest that this actin structure is actively assembling/disassembling leading to catastrophe when the drug blocked elongation.

We found that the actin nucleation factor cortactin was abundantly localized to the distal fungipod. Interestingly, cortactin was configured in a conical sheath with a core rich in F-actin (Figure 3D, E). The fungipod/zymosan contact site viewed *en face* revealed a ring of cortactin surrounding an actin core. Since cortactin is associated with the generation of Arp2/3-mediated dendritic actin networks, these data suggest that a dense branched actin network is generated in the distal fungipod.

We next examined the involvement of microtubules in fungipods. Immunofluorescence localization of α-tubulin revealed normal microtubular staining in the DC cell body (Figure 3F) but a diffuse tubulin distribution throughout the fungipod (Figure 3G). We observed no microtubules in distal or proximal fungipods by immunofluorescence or TEM thin sections (Figure 1F; data not shown). Furthermore, treatment of existing fungipods with the microtubule depolymerizing drug nocodazol had no effect on fungipod structural integrity or growth even after prolonged exposure (Figure 3H). Therefore, we conclude that the cytoskeletal structure of DC fungipods is actin-driven and not dependent upon microtubules.

### Fungipodial Dynamics

**Figure 4:**
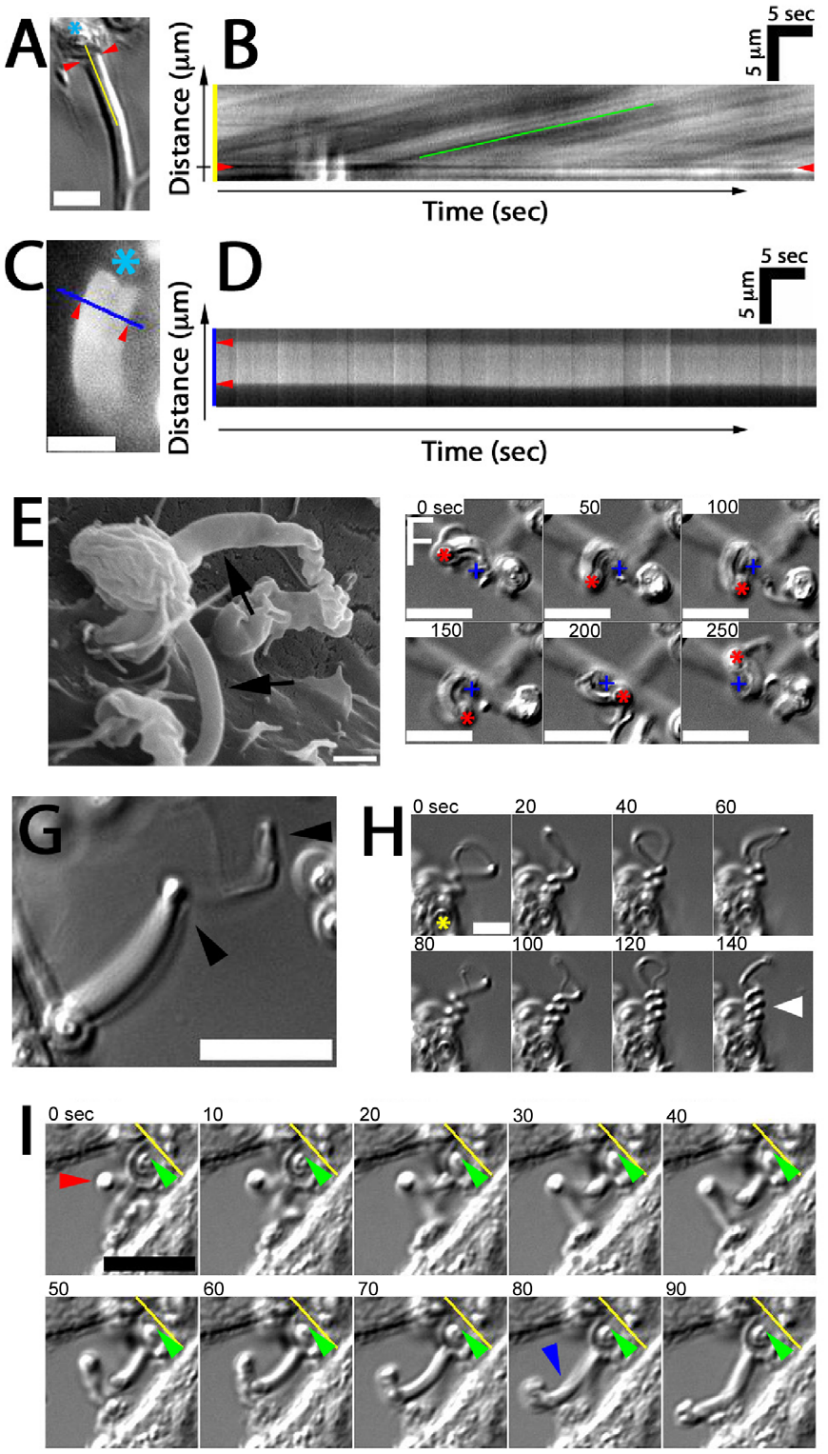
Fungipod dynamics. (A) Representative DIC image showing the zymosan particle (asterisk), contact site (arrowheads) and line used for kymograph analysis. Bar = 5 µm. (B) Representative kymograph showing DIC intensity along the line from the previous panel over time. Rearward flow appears as diagonal lines and speed of flow is measured from the slope of these lines. (C) DiI labeled distal fungipod overlaid with a line indicating the region used for kymography to observe changes in the fungipod edge position over time. Asterisk indicates position of attached zymosan. Bar = 5 µm. (D) Kymograph showing non-undulating edges of DiI stained distal fungipod. Arrowheads mark the edges of the fungipod. (E) SEM image of distal fungipods with arrows emphasizing the smooth lateral edges of these structures. Bar = 1 µm. (F) DIC time series showing rotation of a zymosan particle attached to a fungipod. The crosses and asterisks indicate the positions of the site of proximal fungipod attachment to the cell body and the zymosan particle, respectively. Bar = 10 µm. (G) DIC image of a fungipod showing a kinked appearance (arrowheads) developed during growth of the structure. Bar = 10 µm. (H) DIC time series showing supercoiling of a fungipod (arrowhead) attached to the zymosan particle indicated by an asterisk. Bar = 5 µm. (I) DIC time series showing a kinked fungipod (red arrowhead) associated with motion of the attached zymosan into the cell membrane as shown by the yellow reference line and green arrowhead. Blue arrowhead emphasizes relaxation of the kinked fungipod. Bar = 10 µm.

As previously mentioned, elongating fungipods were often apparent in DIC time series. Upon closer examination we noted that a rearward flow of refractile material moving from the contact site toward the cell body was visible in the distal fungipod (Video S4). Kymographic velocity analysis revealed an apparent rearward flow of 225±55 nm/second (N = 40 velocity measurements in 8 fungipods; Figure 3A, B). We considered that mobile ripples on the membrane could cause a spurious appearance of flow, so we stained DC membranes with DiI and observed distal fungipod membranes for undulations. We saw no undulation of fungipod membranes in movies acquired at ∼7 Hz (Figure 4C, D), and we also note that distal fungipod membranes appear quite smooth by SEM imaging (Figure 4E).

We also observed that fungipodial growth is accompanied by rotation about the longitudinal axis of the fungipod. In cases where the attached zymosan particle became detached from the cell surface while still bound at the fungipod contact site, the fungipod particle was observed to rotate or be driven in a circular motion (Figure 4F; data not shown). In more constrained cases this rotation resulted in kinking and even supercoiling of the fungipod (Figure 4G, H; Video S5). The function of fungipod growth with rotation is not completely clear. We have observed fungipod kinking coincident with visible and repeated displacement of the attached zymosan into the cell membrane (Figure 4I; Video S6) followed by eventual phagocytosis of the particle. One possible interpretation is that fungipod rotation during growth results in a twisted fungipod that upon relaxation applies a force displacing the zymosan forward (i.e., toward the DC membrane) thus promoting particle retention and perhaps aiding in phagocytosis.

### C-type Lectins and Generation of Fungipods

**Figure 5:**
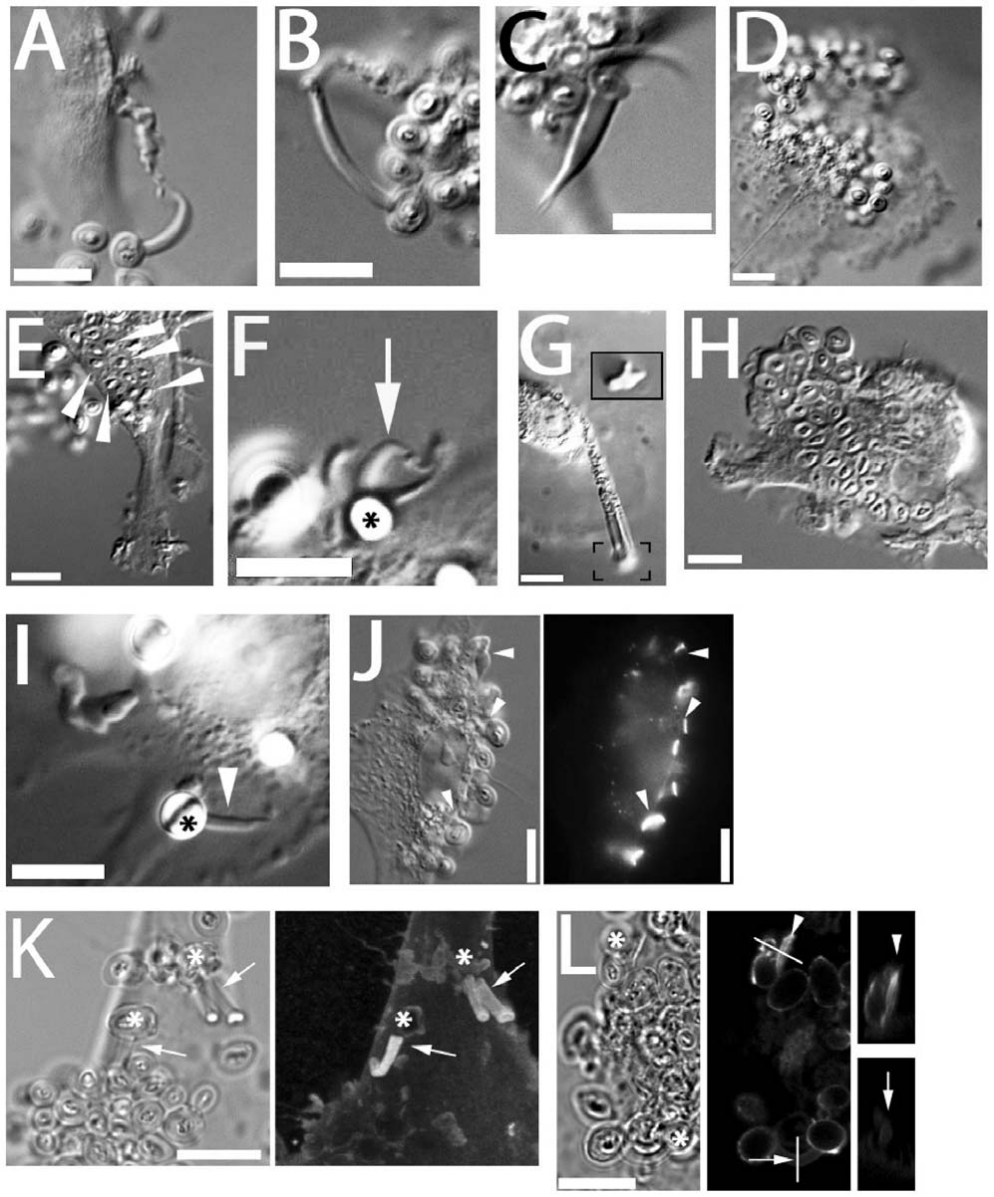
C-type lectin receptors and generation of fungipods. (A) Laminarin (5 mg/ml) blocked DC produced normal fungipods. (B) Anti-dectin-1 polyclonal antibody (10 µg/ml) blocked DC produced normal fungipods. (C) Pharmacological inhibition of Dectin-1 signaling with Syk inhibitor II (1 µM) does not block formation of normal fungipods. (D) Blocking with soluble mannan (5 mg/ml) does inhibit the formation of fungipods although DC-associated zymosan particles are still observed. (E) Internalized zymosan particles (arrowheads) are still observed in the presence of blocking with soluble mannan. (F) Mannan-coated 5 µm beads (asterisk) induced protrusions (arrow) identical to fungipods. (G) Chitin particles induced protrusions identical to fungipods. Inset contains the bracketed area reproduced at 2× higher magnification than the main panel and focused at a lower plane to allow imaging of the chitin particle. (H) Blocking with anti-CD206 polyclonal antibody (50 µg/ml) abolished formation of zymosan induced fungipods but did not prevent binding to DC or internalization. (I) Anti-CD206 antibody coated 5 µm beads induced the formation of protrusions identical to fungipods. (J) DIC (left panel) and epifluorescence (right panel) imaging of intense CD206 staining at zymosan-DC contact sites (arrowheads). (K) DIC (left panel) and confocal fluorescence (right panel) imaging of clathrin light chain in zymosan (asterisks) induced fungipods. Right panel is a maximum intensity projection in the z-axis of a three dimensional confocal stack. Arrows indicate fungipods bearing clathrin LC. (L) DIC (left panel) and confocal fluorescence (middle and right panels) imaging of dynamin in DC/zymosan (asterisks) contacts. Lines denote locations of orthogonal sections through a membrane ruffle near a zymosan particle (arrowhead) and a mature fungipod (arrow). Orthogonal sections are shown in the right panels. Bar = 10 µm.

We tested whether ligation of the phagocytic receptor Dectin-1 by zymosan was involved in the generation of fungipodial protrusions. The β-glucan laminarin binds Dectin-1 and inhibits its interaction with zymosan particles. We found that blocking with excess soluble laminarin did not inhibit the formation of zymosan-induced fungipods or number of zymosans per cell (Figure 5A; Figure S2A, B). Similarly, blocking with anti-Dectin-1 polyclonal antibody did not prevent formation of fungipods (Figure 5B). Anti-Dectin-1 blocking did reduce the amount of internalized zymosan (Figure S2C) consistent with its known role in yeast phagocytosis [32],[33]. Finally, pharmacological inhibition of Syk (activated by Dectin-1) also did not block fungipod formation (Figure 5C). Together these data indicate that Dectin-1 is not required for fungipod generation.

Treatment with excess soluble mannan completely inhibited formation of fungipodial protrusions (Figure S2B). Mannan blocking also reduced the number of cell-associated and internalized zymosans per cell (Figure S2A, C), consistent with prior reports [34]. However, extracellular membrane-associated particles were still seen (Figure 5D) and examples of internalized zymosan particles (presumably via Dectin-1) further proved that zymosan particles were able to interact with DC membrane proteins under mannan blocking conditions (Figure 5E). Furthermore, mannan blocking greatly reduced fungipod formation efficiency (Figure S2D) indicating that inhibition of fungipods by mannan was due to abolition of a mannan-sensitive receptor interaction and not merely by the decrease in binding. This blocking experiment implicated receptors with affinity for high-mannose polysaccharides (i.e., DC-SIGN and CD206) in the generation of fungipods.

We adsorbed mannan to 5 µm polystyrene beads and exposed DC to these beads. We found that bead-immobilized mannan was sufficient to generate fungipods with similar size and structure to those observed after zymosan exposure (Figure 5F). In addition, we found that particles of purified chitin similar in size to zymosan particles (∼5 µm) were also capable of generating fungipods with a normal appearance and an apparent rearward flow visible in DIC (Figure 5G; Video S7). As a negative control for non-specific effects of particulate binding to DC surfaces, we coated 5 µm beads with bovine serum albumin, chicken ovalbumin or fetal bovine serum proteins and applied these beads to DC. While the beads were able to attach to the DC, no fungipods were induced by these non-specific control beads (Number of bead-bound cells observed: BSA, N = 56; OVA, N = 57; serum, N = 54). As a further specificity control, we applied Alexafluor-488 labeled *E. coli* to DC for 4 hours. No fungipods were present at the end of this period and the bacteria were entirely internalized at this point (data not shown). We concluded that the receptor responsible for fungipod generation possesses binding affinity for both mannan and chitin. CD206 has these characteristics whereas DC-SIGN does not bind chitin. Blocking with anti-DC-SIGN polyclonal antibody failed to significantly reduce fungipod numbers or the efficiency of their formation (Figure S2B, D) further demonstrating that DC-SIGN/zymosan interaction is not required for fungipod growth.

Blocking CD206 with anti-CD206 polyclonal antibody prior to addition of zymosan dramatically inhibited formation of fungipods and the efficiency of their formation (Figure 5H; Figure S2B, D). Anti-CD206 antibodies adsorbed on 5 µm beads induced abundant fungipods with normal morphology (Figure 5I). CD206 crosslinking with soluble secondary antibody did not induce fungipods (data not shown). In summary, when CD206-mediated interactions between the DC and zymosan are blocked by soluble antibody, the fungipod fails to form. Conversely, when an antibody-coated, zymosan-sized bead engages CD206, a fungipod is generated. Therefore, we conclude that the C-type lectin receptor CD206 is necessary and sufficient for the formation of zymosan-induced fungipods on immature DC.

CD206 was greatly enriched at most zymosan/DC contact sites including the contact site on the distal fungipod (Figure 5J). CD206 is known to associate with clathrin via AP-2 interactions with its cytoplasmic tail, and we found that clathrin light chain was abundant throughout the distal fungipod (Figure 5K). Dynamin can be recruited to clathrin patches and contributes to initiating actin reorganization. Therefore, we investigated dynamin localization in zymosan-apposed membranes. While mature fungipods were dim or negative for dynamin staining, we found smaller membrane protrusions juxtaposed to zymosan that stained intensely for dynamin (Figure 5L).

### Functional Significance of Fungipods

**Figure 6:**
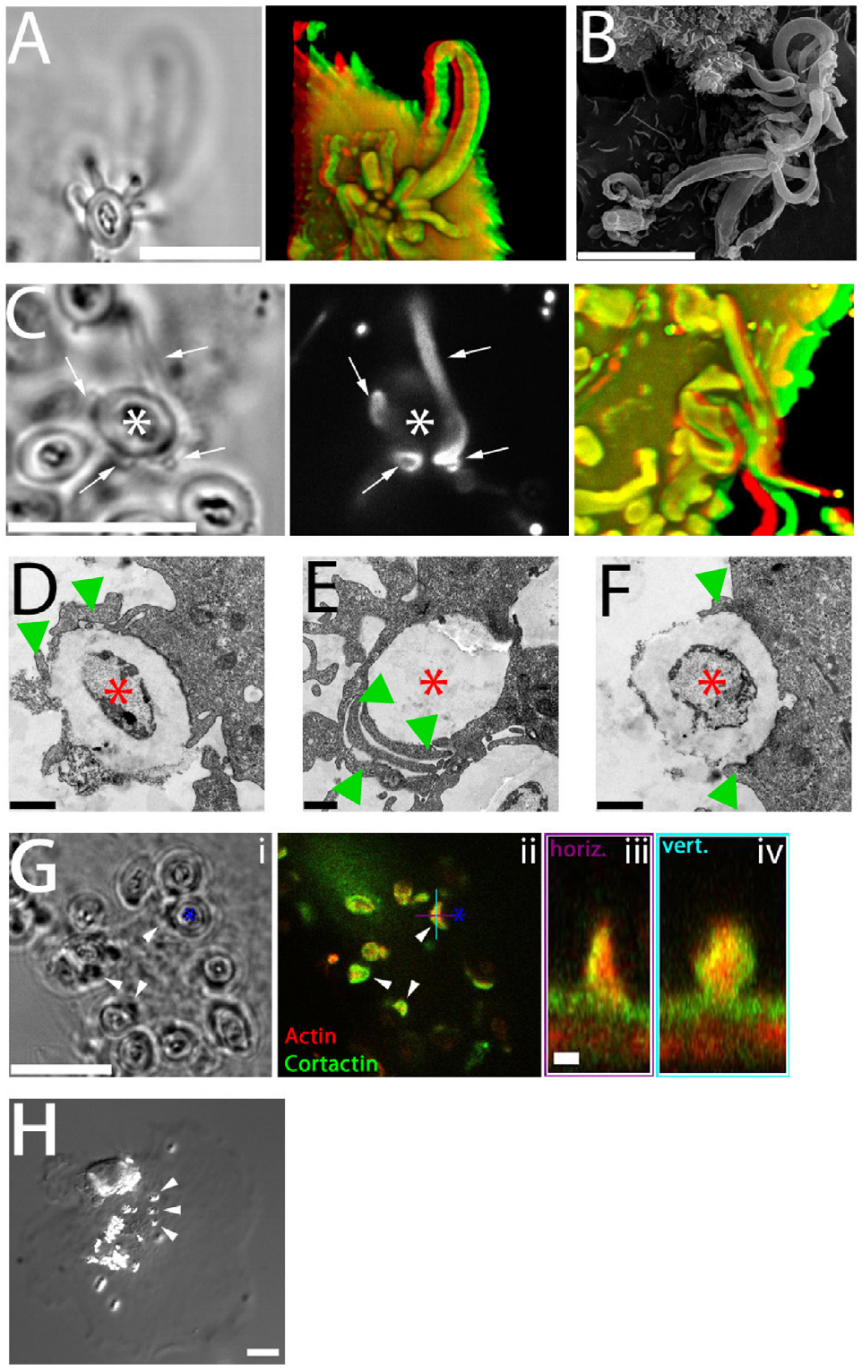
Functional significance of fungipods. (A) DIC (left panel) and confocal 3D projection (right panel, red/green anaglyph) images of actin staining in DC with a zymosan particle attached in a cup-like structure formed by 14 fungipods. Zymosan position is shown in DIC. Bar = 10 µm. (B) SEM image (3700x) of zymosan particles with numerous attached fungipods. Bar = 10 µm. (C) DIC (left panel), confocal fluorescence (middle panel), and confocal 3D projection (right panel, red/green anaglyph) images of DiI stained DC with fungipods (arrows) enmeshing a zymosan particle (asterisk). Bar = 10 µm. (D) TEM thin section image of a zymosan particle (asterisk) being monolaterally engulfed (arrowheads) by a DC. Bar = 1 µm. (E) TEM thin section image of an internalized zymosan particle (asterisk) associated with overlapping phagocytic membranes (arrowheads). Bar = 1 µm. (F) TEM thin section image of a zymosan particle (asterisk) being bilaterally engulfed (arrowheads) by a DC. Bar = 1 µm. (G) DIC (i) and confocal fluorescence (ii) images of zymosan (asterisk) associated membrane wedges on DC (arrowheads) stained for actin (red) and cortactin (green). Orthogonal sections of a membrane wedge through horizontal (purple) and vertical (cyan) lines shown are depicted in panels (iii) and (iv), respectively. Bar = 10 µm for panels (i, ii). Bar = 1 µm for panels (iii, iv). (H) Representative DIC image of anti-CD206 coated 1 µm beads (arrowheads) bound to DC for 4 hours resulting in no fungipod-like protrusions. Bar = 10 µm.

We have noted that fungipod/yeast attachments appear quite durable as we do not observe loss of yeast particles over hours of imaging and the contacts are quite tightly apposed. Furthermore, we often saw single zymosan particles contacted by multiple distinct fungipods (as many as 14) (Figure 6A, B; Video S8). We also observed that zymosan particles could become wrapped within a cage of membrane protrusions formed by lateral interactions of the proximal and distal fungipod walls with the zymosan particle (Figure 6C; Video S9). Such tethering via multiple attachments is likely to provide extremely stable retention of bound zymosan particles thus improving engulfment and antigen sampling for immature DC.

To examine the kinetics and probability distribution of internalization and/or fungipod formation, we have undertaken an extensive quantitative analysis of individual zymosan histories (N = 301) as observed in their interactions with DC during a cumulative ∼980 hours in contact with DC. We quantified the distribution of zymosan in various states (DC surface bound, fungipod-associated, and internalized) as well as the kinetics of transition between those states (summarized schematically in Figure S3A). When direct internalization (without a fungipod) was observed, it occurred at an average time of 72±58 minutes. When fungipod formation occurred, it was seen at an average time of 78±57 minutes. Internalization of a fungipod-associated zymosan required an average time of 160±111 minutes (Figure S3B). These kinetic measurements were based on the actual time that an individual zymosan particle took to make the indicated transition between states (i.e., time from initial binding of a zymosan until a fungipod was first seen associated with that particle). The movies used to obtain this kinetic information varied in length but ∼90% were >160 minutes duration (movie durations in minutes: minimum, 85.2; maximum, 975; mean, 368; standard deviation, 210). Zymosan particles frequently remained bound to the DC surface for many hours without fungipod formation or internalization accounting for 63.9% of the zymosans observed. Direct internalization was inefficient accounting for only 14.5% of bound zymosans. Of the remaining surface bound zymosans, another 21.6% made fungipods. Of these fungipod-associated zymosans, 14.6% were internalized (Figure S3C). The ∼20% of surface-bound zymosans that formed fungipods were non-productive for direct phagocytosis from the plasma membrane.

Coiling phagocytosis has been described in the internalization of *Legionella pneumophila* [35] and also for fungal particles [36],[37]. This process is morphologically defined by the presence of monolateral phagocytic membrane engulfment and overlapping phagocytic membrane extensions seen in thin section TEM rather than classical bilateral phagocytic engulfment [38]. We have documented both of these morphologies in TEM thin sections of zymosan attached to DC (Figure 6D, E) in addition to apparent bilateral engulfment (Figure 6F). Interestingly, we also find that zymosan particles are frequently associated with smaller wedge-like projections of membrane that contain actin and cortactin (Figure 6G; Video S10). These wedge structures are consistent with the expected monolateral engulfment in coiling phagocytosis and were observed with or without coincident fungipods.

While anti-CD206 coated 5 µm beads do generate fungipods, 1 µm beads coated at the same surface density of anti-CD206 are not capable of generating fungipodial protrusions despite avid binding to the DC membrane (Figure 6H). This result implies that the DC can distinguish between particles with identical composition but different size, and fungipodial protrusions are only generated as part of a response against large particles.

### Fungipod Response to *Candida* Yeasts

**Figure 7:**
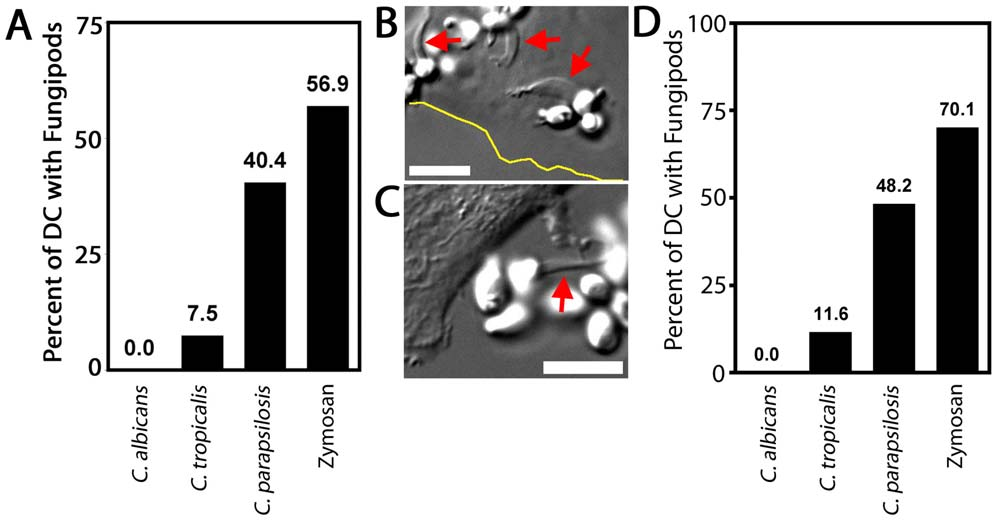
Fungipods and *Candida* species. (A) Percent of DC possessing surface-bound live yeast particles that made fungipods after 4 hour, 37°C DC co-culture with *C. albicans* (N = 82), *C. tropicalis* (N = 67), *C. parapsilosis* (N = 47) or zymosan (N = 65). (B) DIC image of *C. parapsilosis* on the dorsal surface of a DC (cell edge shown by yellow line) with associated fungipods (red arrows). (C) DIC image of *C. tropicalis* attached to a DC via a fungipod (red arrow). (D) Percent of DC possessing surface-bound fixed yeast particles that made fungipods after 4 hour, 37°C DC co-culture with *C. albicans* (N = 138), *C. tropicalis* (N = 129), *C. parapsilosis* (N = 114) or zymosan (N = 117). Bars = 10 µm.

We co-cultured DC with log-phase live *Candida* species yeasts for four hours and measured the percentage of cells producing fungipods in response to surface bound *Candida* or zymosan (as a positive control) (Figure 7A). As previously shown, zymosan exposure evoked a strong fungipod response with 56.9% of particle bearing DC responding (N = 65). *C. parapsilosis* also triggered strong fungipod formation with 40.4% of DC responding (N = 47) (Figure 7B). *C. tropicalis* binding to DC resulted in fungipod formation only rarely (7.5% responding, N = 67) (Figure 7C). We observed no fungipods on DC bound by *C. albicans* (N = 82). Phagocytosed *C. albicans* persists in the phagosome and forms intracellular pseudohyphae that can grow inside phagocytes leading to their destruction [39]. This raised the possibility that *C. albicans* might induce fungipods but the interacting DC might be destroyed prior to four hours of co-culture. We similarly failed to observe fungipods in DC/*C. albicans* co-cultures of one or two hours duration (data not shown), suggesting that DC lysis does not explain the lack of fungipods formed in response to *C. albicans*. To test whether *Candida* species yeasts might be actively encouraging or inhibiting fungipod formation (i.e., through secreted factors), we fixed early log-phase cultures of the three *Candida* species and applied these yeast particles to DC. These fixed particles were almost entirely composed of yeast-form cells. The identical trend in fungipod production was the same for fixed *Candida* as for live cells (Figure 7D). Some increased responsiveness in the fixed *Candida* experiments was observed, but this was a global effect and is likely explained by the use of different monocyte donors in these two sets of experiments. The similarity in trends between live and fixed yeasts suggests that differential fungipod responses to *Candida* pathogens is due to intrinsic yeast characteristics, not acutely active microbial processes that influence fungipod biogenesis.

## Discussion

We have described a novel protrusion, termed the fungipod, that is produced on immature DC by fixed and live *S. cerevisiae* and *Candida* species. They originate from the dorsal DC membrane and they consist of a slender, convoluted proximal region and an ordered distal region. The distal fungipod tip is tightly apposed to the particle, which often remains independently associated with the DC plasma membrane via another contact site. The distal fungipod consists of a sheath of cortactin surrounding a central density of F-actin, and the fungipodial protrusion is sensitive to cytochalasin D. Microtubules are not required for fungipods. The F-actin structure in the distal fungipod exhibited 225±55 nm/second rearward mobility visible by DIC. We found that ligation of the C-type lectin CD206 is the necessary and sufficient condition for generation of fungipodial protrusions, and we observed a significant concentration of CD206 at zymosan contact sites. Clathrin light chain was abundant in mature fungipods, and dynamin was present in small zymosan apposed membrane protrusions but not mature fungipods. Fungipods may help retain fungal particles on the cell membrane and are coincident with hallmarks of coiling phagocytosis on DC. Fungipods discriminate particle size since yeast-sized 5 µm, but not 1 µm, CD206 ligand-conjugated beads generate fungipods.

### The Fungipod/Yeast Interface

The distal fungipod's close contact site with the zymosan particle often contained regions of greater electron density along the apposed membrane visible in TEM thin sections (Figure 1F, G). We also observed these membrane densities on zymosan-containing phagosome walls. Interestingly, the densities displayed a clustered, knobbed pattern projecting outward from the cell. The DC membrane is strongly enriched in CD206 at the zymosan contact site (Figure 5J). Therefore, the membrane densities observed by TEM at this site may represent concentrations of CD206, perhaps in membrane microdomains as has been observed previously for another transmembrane C-type lectin, DC-SIGN [7].

### Triggering the Fungipod: CD206 and Actin Reorganization

The cytoplasmic domain of CD206 contains a tyrosine-based internalization motif responsible for mediating endocytosis via clathrin coated pits [18]. Indeed, we have observed pits at the fungipod/zymosan contact and strong clathrin light chain localization in distal fungipods (Figure 1G; Figure 5K). CD206-driven clathrin patches may assemble at zymosan contact sites. Dynamin recruitment to clathrin patches coordinates actin polymerization involved in vesicle scission and propulsion into the cytoplasm [29],[30],[31]. Frustrated clathrin-mediated endocytosis may generate a signaling platform as hypothesized below.

Dynamin can recruit cortactin to the membrane leading to actin polymerization. In addition to this localization, cortactin activity can be regulated by Src-family kinases (SFK) [21]. Neither the SFK inhibitor PP2 nor the broad-spectrum protein tyrosine kinase inhibitor genistein inhibited fungipods suggesting that cortactin tyrosine phosphorylation is dispensable for fungipod biogenesis (data not shown). Cortactin activity does not always require tyrosine phosphorylation (i.e., for actin pedestal formation) [23].

**Figure 8:**
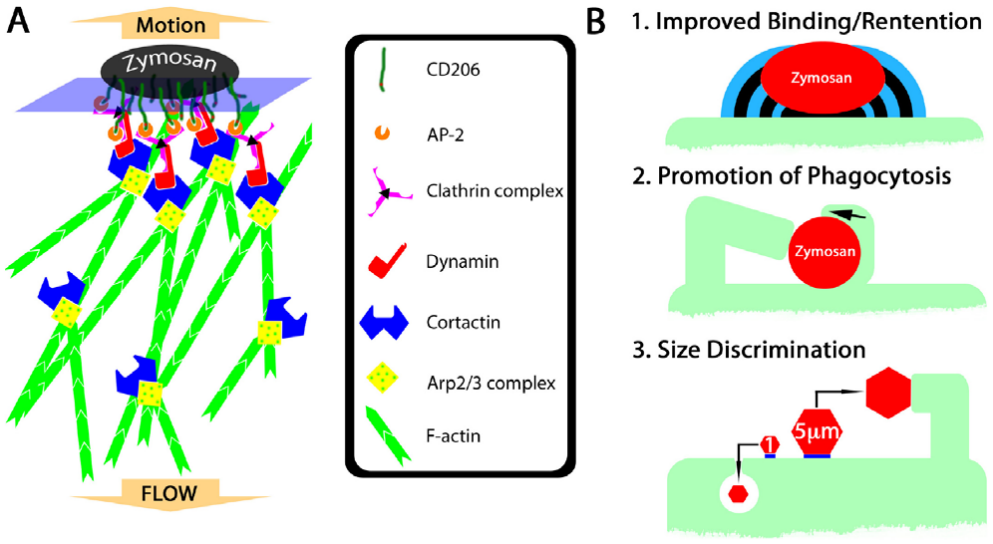
Hypothetical mechanism of fungipod formation and functional roles of fungipods. (A) Hypothetical protein complex involved in initiation and growth of fungipods. CD206 recruits clathrin complexes via AP-2 interactions with its tyrosine-based internalization motif. Clathrin binds dynamin, which can recruit cortactin leading to stabilization of Arp2/3 complexes and elongation of F-actin in dendritic networks. Cortactin also stabilizes actin branch points permitting greater longevity and stiffness of the F-actin network. Steady state polymerization of actin at the zymosan-proximal tip results in rearward flow of actin and a reaction force pushing the zymosan particle outward. (B) Summary of potential functional significances of fungipods for DC biology. (1) Fungipods may improve binding and retention of large particles such as zymosan that would be easily removed by shearing contact with other cells. (2) Fungipods may assist in phagocytosis (including monolateral engulfment/coiling phagocytosis) of yeast particles by holding this large particle in place during engulfment. (3) Fungipods may be part of the DC's specific size discriminatory response program directed against large (i.e., 5 µm) particles, but not smaller particles (i.e., 1 µm) that are more easily dealt with through conventional phagocytic means.

Consistent with the hypothesis that CD206 concentration at zymosan contact sites catalyzes the development of clathrin patches leading to dynamin recruitment, we have observed dynamin enrichment in small membrane protrusions next to zymosan particles. Since dynamin binds cortactin [31] and this promotes actin polymerization, the size and duration of clathrin/dynamin/cortactin complex recruitment might be important in driving the formation of fungipods (Figure 8A). Finally, cortactin's ability to stabilize Arp2/3 branch points [19] and bundle actin filaments [40],[41] may influence the durability and stiffness of dendritic actin networks in the distal fungipod. We have summarized our data regarding the mechanism of fungipod formation (Figure S4A).

Heinsbroek, et al have observed a sequential engagement of C-type lectins by zymosan and *C. albicans* in murine thioglycollate-elicited macrophages [42]. They reported that Dectin-1 was responsible for immediate binding and internalization of fungal particles while CD206 associated only later with phagosomes leading to MCP-1 and TNF-α production. Our experiments in a different species and cell type have shown that zymosan acquisition by human immature DC is highly dependent on CD206 as binding is blocked by mannan and anti-CD206 (Figure S2A). However, this early role for CD206 in our system does not preclude a later role in signaling from accumulations of CD206 in phagosomes or fungipod-associated membranes. An important caveat in this comparison with Heinsbroek, et al is that we have not observed fungipods on murine bone-marrow derived immature DC stimulated with zymosan or *Candida* species, and murine immature DC phagocytose zymosan approximately 1–2 orders of magnitude faster than their human counterparts (data not shown). Rapid internalization in murine cells may not allow sufficient time or available membrane surface area for fungipod extension. Apparent differences between murine and human DC handling of yeast particles suggests that there may be significant underlying differences in the function of and signaling by C-type lectins between mouse and human DC.

### Similarity to Actin Rocketing Systems

Several features of the zymosan-induced fungipods described in this report bear similarity to actin comet tails associated with *Listeria monocytogenes*, *Shigella flexneri*, vaccinia virus and rocketing vesicles [43],[44],[45]. The overall size and shape of fungipodial actin structures as well as their propensity to elongate with rotation are mirrored by tapered F-actin comet tails that arc in either right or left handed fashion through the cytoplasm behind motile intracellular pathogens [46]. The speed of *Listeria* or ActA-coated bead propulsion is roughly similar to our report of 225±55 nm/second for fungipodial rearward flow [47]. The rearward mobile bands visible by DIC in the distal fungipod are similar to actin densities in comet tail bands seen behind larger (>3 µm) actin rocketing beads [48]. The distal fungipod contains copious amounts of cortactin, which is also found in actin comet tails. Interestingly, *Listeria* actin comet tails contain cortactin throughout but do not contain phosphotyrosine epitopes in the tail suggesting lack of cortactin tyrosine phosphorylation as another point of similarity between fungipods and *Listeria* comet tails [49]. This similarity of structure, composition and behavior may point to underlying similarities in mechanism of formation between actin comet tails and fungipods.

### Actin Transport and Polymerization in the Distal Fungipod

We asked whether our observations of growth in the distal fungipod are comparable to what is understood about actin polymerization and actin rocketing systems in vivo and in vitro. Our measurements of rearward flow in the distal fungipod imply a dendritic actin growth speed of ∼200 nm/second. Assuming actin growth at the distal tip in a 1 µm diameter circular contact with filament barbed ends placed at 40 nm intervals [50],[51],[52] and monomer unit elongation length of 2.7 nm [53], our rearward flow rate requires polymerization of ∼3.6×10^4^ actin monomers/second at the tip. Previous reports have provided the following information: cytoplasmic G-actin concentration of 12 µM, ATP-actin barbed end assembly k_on_ = 11.6 µM^−1^ second^−1^ and disassembly k_off_ = 1.4 second^−1^ [54],[55]. Given these values and assuming the same polymerization surface described above, “free running” actin assembly/disassembly could achieve “free running” rates of ∼6.8×10^4^ actin monomers/second. Thus, actin polymerization rates required to support the growth of the distal fungipod are in the range of what is possible.

### Functional Significance of Fungipods

What could be the functional relevance of fungipods to DC function and microbe internalization in general? On the basis of our observations, we propose the following areas of functional significance: 1) Improved binding/retention of particles, 2) Promotion of phagocytosis (including coiling phagocytosis), 3) Participation in a size discrimination program in phagocytes (Figure 8B; Figure S4B).

Fungipodial protrusions may promote phagocytosis of yeasts by improving long-term retention. DC experience long contact times with zymosan particles prior to internalization, and many bound particles are not phagocytosed despite hours of contact (Figure 1K; Figure 4I; Video S6; Figure S3A). It is attractive to speculate that fungipods might be rescuing some fraction of non-internalized, surface-bound zymosans from their most likely fate of non-productive surface-association with the DC. This could occur if the surface of the yeast particle was non-uniformly stimulatory for phagocytosis and interaction with a fungipod could reposition the particle or allow improved exploration of its surface by the DC (i.e., via the membrane wedges we have observed in Figure 6G). Our analyses show that zymosans attached to fungipods have a probability of internalization equivalent to that of their non-fungipod associated counterparts (Figure S3C). Because of this, it is possible that fungipods do not influence phagocytosis or even that they delay, but do not absolutely prevent, phagocytosis. Distinguishing between these competing interpretations would help to elucidate the functional role of fungipods, but it will require future development of experimental approaches to specifically ablate fungipods without influencing phagocytosis.

Previous reports have identified “coiling phagocytosis”, a monolateral engulfment that acts to internalize bacteria and yeast via a single pseudopod that wraps around the particle and creates a phagosome. We have identified examples of coiling phagocytosis occurring under conditions where fungipods are present (Figure 6E, F). Of course, we cannot be sure from thin section TEM data that these particular zymosan particles were associated with fungipods. Furthermore, we often see wedge-shaped membrane projections associated with surface bound zymosan particles that fit very closely the type of monolateral engulfing structure that one would expect to see in coiling phagocytosis (Figure 6G; Video S10). Consistent with a role in phagocytosis, these membrane wedges are rich in F-actin and cortactin (Figure 6G).

Professional phagocytes such as macrophages and immature DC must be able to recognize and respond to pathogens with a wide range of sizes from viruses of <100 nm diameter to much larger extracellular pathogens (i.e., helminthes, filamentous fungi and *Leishmania* promastigotes) with sizes actually greater than the phagocyte itself. Typically particles >0.5–1 µm diameter elicit phagocytic activity [56] with an optimum at 2–3 µm [57]. While one still observes phagocytosis of zymosan particles in immature DC, they are larger than the optimum particle size. A possible fungipod ontogeny could include generation of fungipodial protrusions from pseudopods or membrane ruffles that are frustrated in their attempts to engulf the large particle for a prolonged period of time. If fact, we do sometimes see small nodules of apparently ruffling membrane next to zymosan particles visible in DIC and these nodules can eventually become fungipods (Video S2).

We found that yeast-sized mannan or anti-CD206 coated beads (5 µm) induced fungipod formation while similar particles of 1 µm diameter did not suggesting that fungipods are part of a size discrimination capability of immature DC. According to our model of fungipod formation (Figure 8A), a patch of clathrin is stabilized by CD206 ligation under a zymosan particle. The particle size dependence of fungipods may represent the integration of this contact site size in the form of the number of effector proteins (i.e., cortactin) recruited to the contact site. A critical contact site area may exist such that the amount of cortactin recruited may only become sufficient to generate a fungipod once the critical contact area is surpassed. Interestingly, while phosphoinositide 3-kinase (PI3K) is often required for engulfment of large particles (>3 µm), we have found that the PI3K inhibitor LY294002 has no effect on fungipod formation (data not shown).

Pathogenic fungi, such as *C. albicans*, can form large structures that may be difficult or impossible to internalize [58]. Likewise, some nematodes, including parasitic worms, possess chitinous mouthparts and egg shells that could be recognized by CD206 but are clearly too large to engulf. It is interesting to note that recently published intravital imaging of dermal DC interacting with *Leishmania major* promastigotes demonstrated the formation of long pseudopodial DC protrusions contacting promastigotes in vivo [59]. Promastigotes of *Leishmania* species are typically larger than 10 µm, they display mannan on their surface [60], and they interact with innate immune cells via CD206 [61]. Protrusive structures such as the fungipod may be used in innate immune reactions to larger extracellular pathogen structures.

### Fungipod Response to *Candida* Species Yeasts

We observed that DC interaction with *Candida* species leads to fungipod formation suggesting that fungipods are involved in recognition of this medically significant genus of fungal pathogens. However, the fungipod response generated against *Candida* species was clearly dissimilar among species as a robust response occurred against *C. parapsilosis*, a weak response against *C. tropicalis* and no response against *C. albicans*. This specificity is somewhat surprising since *Candida* cell walls are considered to be quite comparable to *S. cerevisiae* cell walls in structure and composition, and the cell wall polysaccharides produced by these three highly related *Candida* yeasts are presumably quite similar. Subtle differences in cell wall polysaccharide structure might underlie the species-specific fungipod response to *Candida*. We observed the same trend of fungipod responsiveness to different species of *Candida* that had been fixed. This rules out active encouragement or inhibition of fungipods by the yeast and is consistent with intrinsic differences in cell wall composition or structure being the cause of the observed differential fungipod response among *Candida* species.

## Materials and Methods

### Monocyte Derived Dendritic Cells (MDDC) and Macrophages

PBMC were isolated from human peripheral blood buffy coats purchased from New York Blood Center (New York, NY). Monocytes were isolated by adherence on tissue culture treated plastic flasks. Immature dendritic cells were prepared by culturing monocytes with 500 U/ml human IL-4 and 800 U/ml human GM-CSF (Peprotech, Rocky Hill, NJ) in RPMI-1640 medium with 10% heat inactivated FBS in glass-bottom MatTek dishes (MatTek Corp., Ashland, MA) for 6 days. Immature macrophages were produced via the same procedure but with omission of IL-4. DC and macrophages were activated with 250 ng/ml LPS (Sigma, St. Louis, MO) for 24 hours prior to use.

### Zymosan/Yeast Particles

Unlabeled, formalin killed zymosan was obtained from Invitrogen (Carlsbad, CA) and resuspended as a PBS stock. Zymosan was used at a final concentration 20 µg/ml (unless otherwise noted) after 3×15 second vortexing (max speed) and 3×15 second bath sonication to achieve monodispersion. Cells were incubated with zymosan for 4 hours, 37°C unless otherwise noted. Live, wild-type *S. cerevisiae* was obtained from log phase cultures and used at comparable density to zymosan. *Candida* species were obtained from the ATCC and grown to log phase culture in YM broth at 30°C then added to DC co-cultures at 5×10^5^ yeast/ml for 4 hours at 37°C. Quantification of fungipods and zymosan was done from 3D image stacks by a single investigator who was blinded to the experimental conditions of these images.

### Fixation for Light Microscopy

Samples prepared for all light microscopic observations except CD206 staining were fixed 20 minutes with 37°C 2.5% glutaraldehyde (Electron Microscopy Sciences, Hatfield, PA) in PBS, pH 7. This fixation provided optimal preservation of fungipod structures, but was not compatible with CD206 immunostaining. For CD206 fluorescence imaging, samples were fixed 20 minutes with 37°C 4% paraformaldehyde (Electron Microscopy Sciences, Hatfield, PA) in PBS, pH 7.

### Wide Field Light Microscopy

Wide field light microscopy was performed on an Olympus IX81 inverted microscope with a 60x, 1.4 NA oil objective lens, stage with z-axis stepper motor control, and objective-based autofocus (Olympus, Center Valley, PA). A 37°C, CO_2_ controlled stage insert (Warner Instruments, Hamden, CT) was used for live cell imaging. DIC images, except those accompanying confocal fluorescence images, were taken using the DIC optical train of this microscope. A 100 W Hg arc lamp provided epifluorescence illumination. Filters and dichroic mirrors (Chroma, Rockingham, VT) for Alexafluor-488 imaging were as follows: excitation, 488/10; emission, 535/25; dichroic, 475/25. For DiI imaging we used the following: excitation, 535/50; emission, 605/40; dichroic, 530/20. Images were captured using an air-cooled SensiCam QE CCD camera (Cooke Corp., Romulus, MI) driven by Metamorph (Molecular Devices, Downingtown, PA).

### Confocal Light Microscopy

Laser scanning confocal microscopy was performed on a Zeiss 510 Meta inverted instrument using a 63x, 1.4 NA oil objective lens. Samples were illuminated with 488 nm and 543 nm lines from a 30 mW Ar ion laser and a 1 mW He-Ne laser, respectively. We used a UV/488/543/633 main dichroic mirror and a NFT545 secondary dichroic in cases of dual color imaging. Alexafluor-488 emission was collected using a LP505 emission filter, and rhodamine fluorescence was collected with a LP560 filter. Data were collected in 1024×1024 pixel format and 12-bit depth, with non-interlaced, descanned, multitracked scanning and 4 scan averaging. Z-axis steps were taken in increments of 200 nm.

### Fluorescent Staining

Primary antibodies used were as follows: cortactin (4F11; Millipore, Temecula, CA), clathrin light chain (CON.1; Santa Cruz Biotechnology, Santa Cruz, CA), CD206 (“anti-hMMR”; R&D Systems, Minneapolis, MN). Antibody staining was done with 10 µg/ml primary antibody, 30 minutes, 25°C. Secondary antibodies (anti-mouse or goat IgG, as appropriate) labeled with Alexafluor-488 (Invitrogen, Carlsbad, CA) were used at 1 µg/ml, 30 minutes, 25°C. F-actin was stained with rhodamine-phalloidin (Invitrogen) according to the manufacturer's instructions. Membrane staining with DiI-C18 (Sigma, St. Louis, MO) was done at 1 µg/ml.

### Scanning Electron Microscopy

Samples were prepared by fixation in 2.5% glutaraldehyde in 0.1 M Cacodylate (pH 7.3) followed by 1% OsO_4_/Cacodylate, dehydration in graded ethanol, critical point drying (CPD 030; Bal-Tec, Vienna, Austria), and sputter coating (Polaron E-5100; Quorum Technologies; East Sussex, UK). Observations were performed using a JEOL 6300 SEM with an Orion Digital Micrography System.

### Transmission Electron Microscopy

Samples were prepared by fixation in 2.5% glutaraldehyde, 1% tannic acid, 0.1 M Cacodylate (pH 7.3), stained with 2% Uranyl acetate, dehydrated in graded ethanol, and embedded in Epon. 60 nm sections were cut and stained with 2% Uranyl acetate followed by Sato lead stain. Observations were performed using a Technai 12 TEM with a Gatan Multiscan 794 digital camera.

### Blocking & Inhibitor Experiments

Blocking reagents were used at the following concentrations: Mannan (10 mg/ml; Sigma, St. Louis, MO), Laminarin (5 mg/ml; Sigma), anti-Dectin-1 polyclonal antibody (10 µg/ml; “anti-hdectin-1/CLEC7A”, R&D Systems, Minneapolis, MN), anti-CD206 polyclonal antibody (50 µg/ml; “anti-hMMR”, R&D Systems). Syk Inhibitor II (EMD Biosciences, Gibbstown, NJ) was used at 1 µM. For blocking and inhibitor experiments, cells were exposed to the agent at 30 minutes, 37°C prior to the addition of zymosan. However, cytochalasin D and nocodazol were used acutely at 1 µM.

### Beads and Chitin Particles

Nominal 5 µm (4.58±0.07 µm) and 1 µm (1.053±0.01 µm) polystyrene beads were obtained from Polysciences (Warrington, PA). Ligands were passively adsorbed on beads using equivalent total surface area of beads in all reactions. After washing with 50 mM bicarbonate buffer (pH 9), adsorption was done in 100 µl total volume (same buffer) at 25°C overnight. For mannan and laminarin, the adsorption reaction concentration was 10 mg/ml. For anti-CD206 polyclonal antibody (R&D systems, Minneapolis, MN) the concentration was 50 µg/ml. For negative controls, beads were coated in 1 mg/ml bovine serum albumin (Sigma, St. Louis, MO), 1 mg/ml chicken egg ovalbumin (Sigma) or neat fetal bovine serum. Beads were washed in PBS and used immediately. Chitin particles (1–10 µm) were prepared by probe sonication and centrifugation as previously described [11], and sizes of individual cell-associated particles were confirmed as ∼5 µm by DIC microscopy.

### Accession Numbers

UniprotKB accession numbers for human proteins referenced in these data are as follows: DC-SIGN (CD209), A8MVQ9; Mannose Receptor (MRC1, CD206), P22897; Dectin-1 (CLEC7A), Q9BXN2; Cortactin, Q96H99; Syk, P43405; Clathrin Light Chain A & B, P09496 & P09497; Dynamin 1 & 2, Q05193 & P50570.

## Supporting Information

Figure S1 Macrophage-produced fungipods and LPS-induced diminution of fungipod formation efficiencies. (A) A human monocyte derived immature macrophage was treated 4 hours with zymosan and fixed. Arrowheads denote zymosans associated with fungipods on a macrophage. Yellow line indicates cell boundary (below focal plane) and bar = 10 µm. (B) Fungipod formation efficiency was calculated for 4 hour zymosan exposed human monocyte derived immature dendritic cells (DC, circles) and immature macrophages (Mac, triangles) untreated (black symbols) or activated with LPS for 24 h prior to zymosan exposure (red symbols). Bars denote average values and statistical significance between untreated and LPS-activated cells (Student's t-test) is provided below the graph. Efficiency was calculated as the number of fungipods per cell divided by number of plasma membrane bound zymosans per cell.
(6.94 MB TIF)

Figure S2 Summary of blocking experiment results. Dendritic Cells were exposed to zymosan for 4 hours either with no treatment or with various blocking reagents as described in Materials and Methods. The following parameters were measured: number of surface bound zymosans per cell (A), number of fungipods produced per cell (B), number of internalized zymosans per cell (C) and efficiency of fungipod formation (D). Red asterisks indicate a significant difference from untreated control at p<0.001, and blue asterisks indicate a significant difference from untreated control at p<0.05. Fungipod formation efficiency was calculated as the number of fungipods per cell divided by number of plasma membrane bound zymosans per cell.
(5.13 MB TIF)

Figure S3 Quantitative analysis of zymosan interaction with dendritic cells with respect to fungipod formation and phagocytosis. (A) Schematic representation of possible zymosan states (“S”, surface bound zymosan; “F”, fungipod associated zymosan; “I”, internalized zymosan) and transitions between states (color coded as shown) that were considered in this analysis. (B) Observed kinetics of state transitions color coded as above. Triangles represent data points where the entire transition was not observable (i.e., zymosan bound prior to start of time lapse, so the exact time of binding was not observed), and thus these are observed minimal times of transition. Circles indicate data points where the entire transition was observed. This data was derived from actual start and end times of transitions between indicated states based on a set of movies with varying lengths (movie durations in minutes: minimum, 85.2; maximum, 975; mean, 368; standard deviation, 210). (C) Probability distribution of zymosan state transitions color coded as above. The upper panel contains the distribution of transitions starting from surface bound zymosan (“S”). This probability was calculated over the entire course of time lapse observation. Of the zymosans that become fungipod associated, further transitions are possible as depicted in the lower panel showing state transitions starting from the fungipod associated zymosan (“F”) state. N = 301 total zymosan particles observed for a cumulative duration of ∼980 hours.
(10.05 MB TIF)

Figure S4 Tables summarizing our findings regarding the mechanism of fungipod formation (A) and perspectives on the functional significance of fungipods (B). Abbreviations: SFK, Src-family kinases; PI3K, phosphoinositide 3-kinase; PTK, protein tyrosine kinase.
(3.58 MB TIF)

Video S1 Zymosan particle attachment and nascence of fungipods on immature DC. Red and green asterisks indicate a stably attached zymosan particle and points of fungipod generation, respectively. Timestamp refers to elapsed time of indicated zymosan particle's attachment. Playback speed is 250x faster than real time. Bar = 10 µm.
(0.36 MB MP4)

Video S2 Maturation of a fungipod formed by an immature DC with attached zymosan particles. Asterisk indicates a relevant zymosan particle. Green and blue arrowheads show nascent and mature fungipods, respectively. Timestamp refers to elapsed time of fungipod maturation. Playback speed is 250x faster than real time. Bar = 10 µm.
(0.67 MB MP4)

Video S3 Three-dimensional projection of actin stained within zymosan induced fungipods on DC showing the robust F-actin signal in the distal fungipod.
(0.08 MB MP4)

Video S4 DIC movie of an apparent rearward flow of refractile material in the distal fungipod. Playback at 10x faster than real time. Bar = 10 µm.
(0.60 MB MP4)

Video S5 DIC time-lapse showing supercoiling of a fungipod. Playback at 250x faster than real time. Bar = 5 µm.
(0.26 MB MP4)

Video S6 DIC time-lapse showing repeated driving of a fungipod-attached zymosan into the DC. The fungipod undergoes repeated cycles of kinking associated with displacements of the zymosan particle into the DC and relaxation of the fungipod. This zymosan is finally phagocytosed by the DC. Playback at 250x faster than real time. Bar = 10 µm.
(1.92 MB MP4)

Video S7 DIC movie of a chitin particle induced protrusion. Playback at 10x faster than real time. Bar = 10 µm.
(0.22 MB MP4)

Video S8 Three dimensional confocal projection of actin staining in a DC with zymosan particle (not visible) attached in a cup-like structure formed by numerous fungipods.
(0.08 MB MP4)

Video S9 Three-dimensional confocal projection of DiI staining in a DC with zymosan particle (not visible) enmeshed by fungipods.
(0.13 MB MP4)

Video S10 Three dimensional confocal projection of actin staining in a DC exhibiting several small zymosan-associated membrane wedges in addition to one large fungipod. Positions of zymosan particles are shown in the DIC insert image (insert bar = 10 µm).
(0.07 MB MP4)
