# Supplementary figures and images for: A Novel Pseudopodial Component of the Dendritic Cell Anti-Fungal Response: The Fungipod
Source: PLoS Pathog. 2010 Feb 12;6(2):e1000760. doi: 10.1371/journal.ppat.1000760 (PMC2820528; doi:10.1371/journal.ppat.1000760)

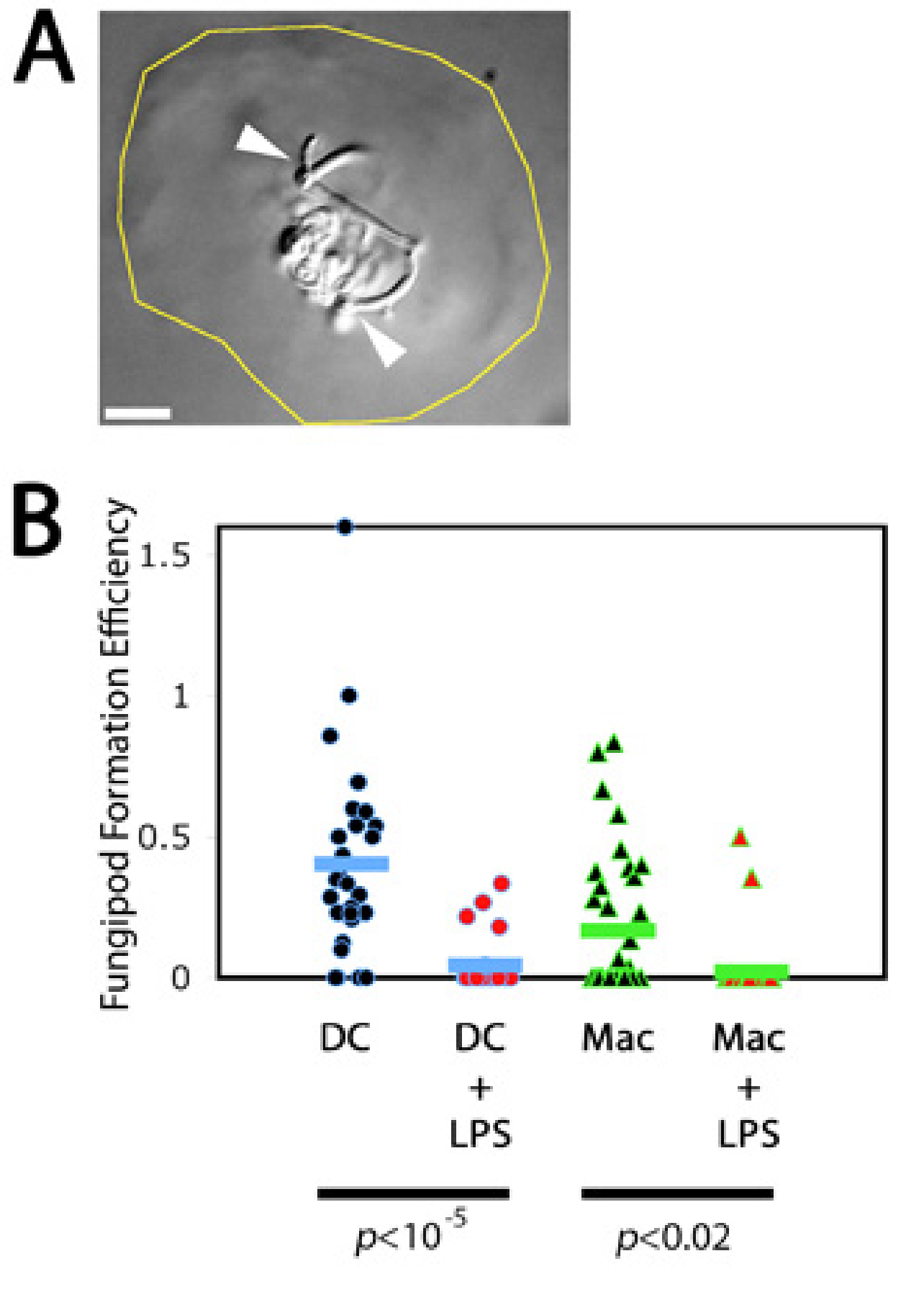

Supplement: Figure S1 — Macrophage-produced fungipods and LPS-induced diminution of fungipod formation efficiencies. (A) A human monocyte derived immature macrophage was treated 4 hours with zymosan and fixed. Arrowheads denote zymosans associated with fungipods on a macrophage. Yellow line indicates cell boundary (below focal plane) and bar = 10 µm. (B) Fungipod formation efficiency was calculated for 4 hour zymosan exposed human monocyte derived immature dendritic cells (DC, circles) and immature macrophages (Mac, triangles) untreated (black symbols) or activated with LPS for 24 h prior to zymosan exposure (red symbols). Bars denote average values and statistical significance between untreated and LPS-activated cells (Student's t-test) is provided below the graph. Efficiency was calculated as the number of fungipods per cell divided by number of plasma membrane bound zymosans per cell. (6.94 MB TIF) [file ppat.1000760.s001.tif]

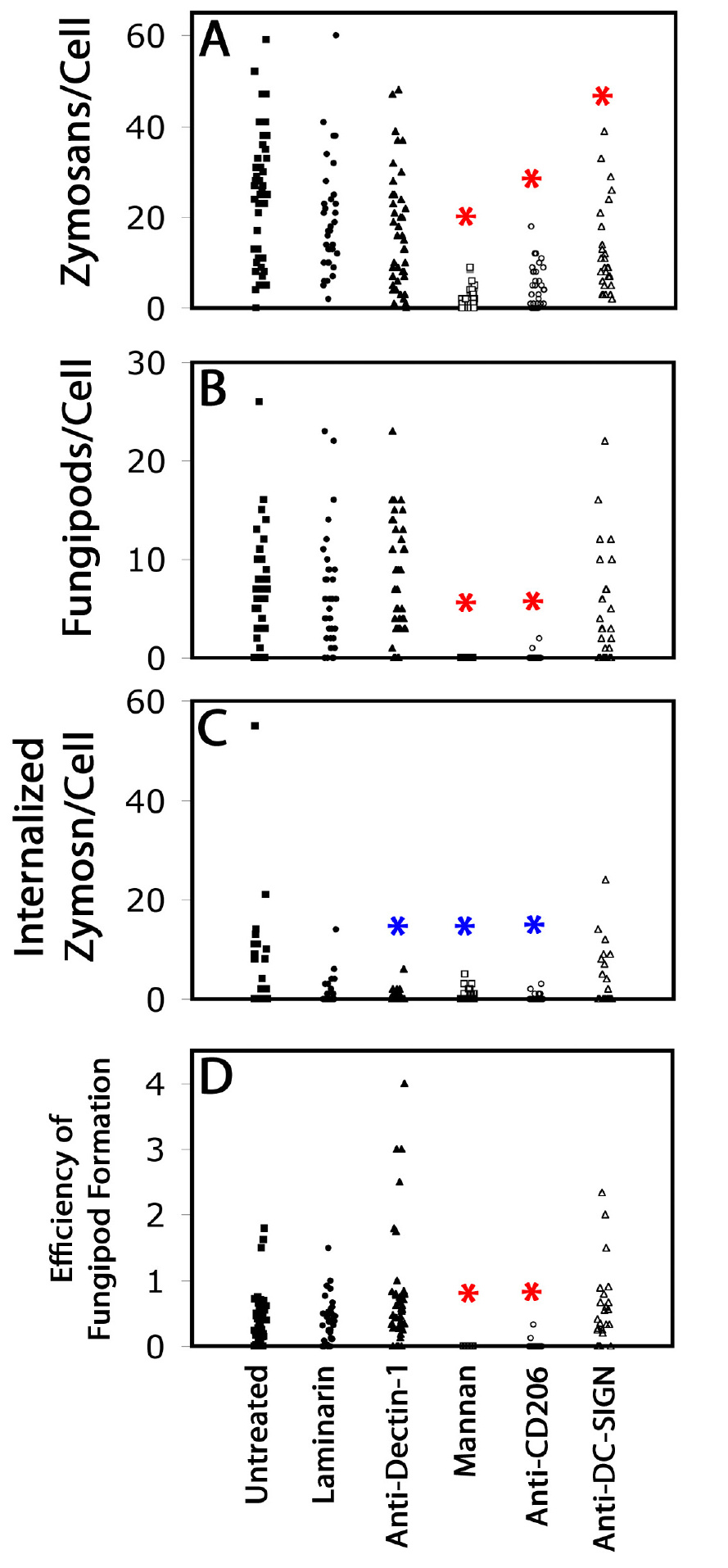

Supplement: Figure S2 — Summary of blocking experiment results. Dendritic Cells were exposed to zymosan for 4 hours either with no treatment or with various blocking reagents as described in Materials and Methods. The following parameters were measured: number of surface bound zymosans per cell (A), number of fungipods produced per cell (B), number of internalized zymosans per cell (C) and efficiency of fungipod formation (D). Red asterisks indicate a significant difference from untreated control at p<0.001, and blue asterisks indicate a significant difference from untreated control at p<0.05. Fungipod formation efficiency was calculated as the number of fungipods per cell divided by number of plasma membrane bound zymosans per cell. (5.13 MB TIF) [file ppat.1000760.s002.tif]

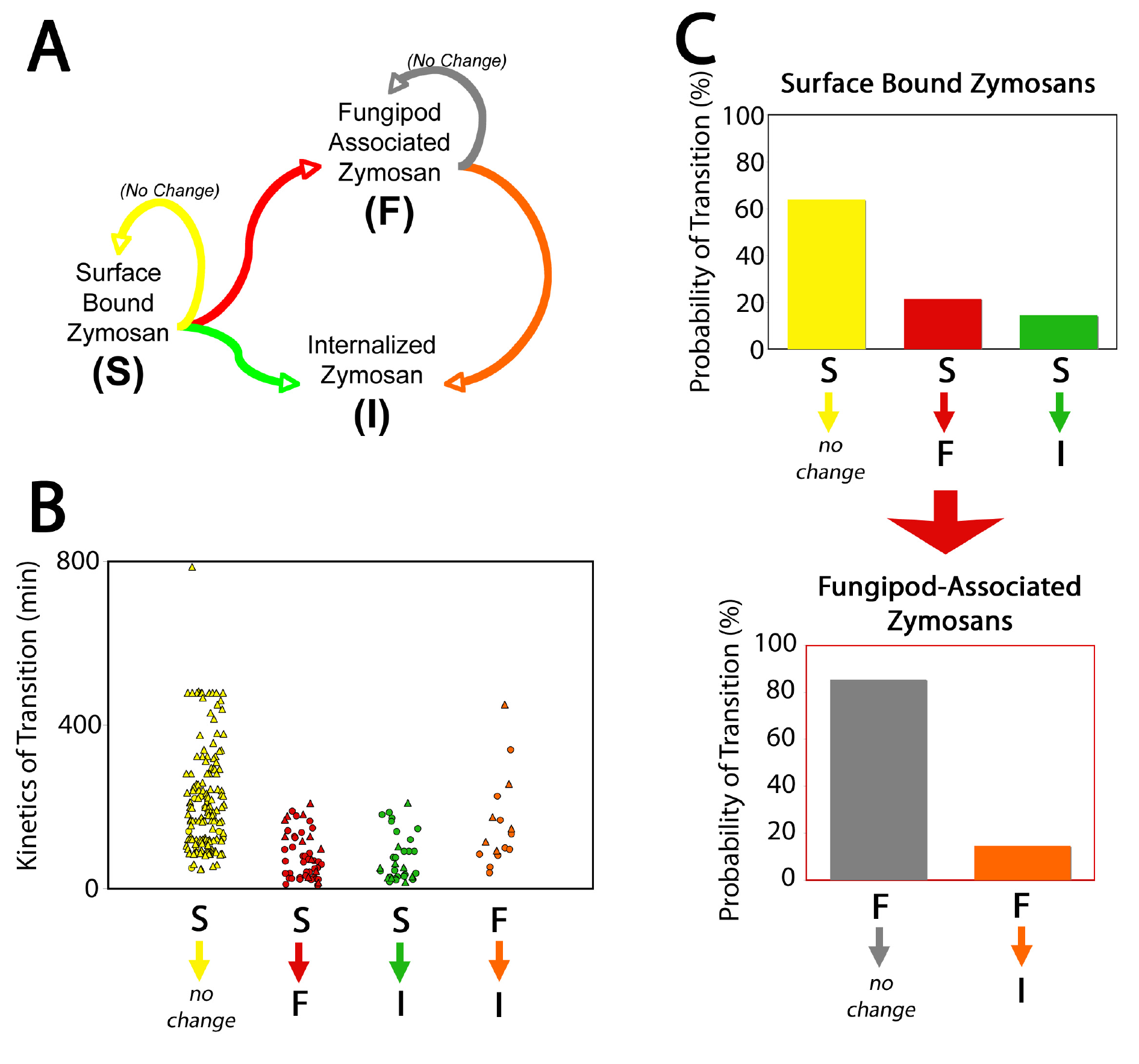

Supplement: Figure S3 — Quantitative analysis of zymosan interaction with dendritic cells with respect to fungipod formation and phagocytosis. (A) Schematic representation of possible zymosan states (“S”, surface bound zymosan; “F”, fungipod associated zymosan; “I”, internalized zymosan) and transitions between states (color coded as shown) that were considered in this analysis. (B) Observed kinetics of state transitions color coded as above. Triangles represent data points where the entire transition was not observable (i.e., zymosan bound prior to start of time lapse, so the exact time of binding was not observed), and thus these are observed minimal times of transition. Circles indicate data points where the entire transition was observed. This data was derived from actual start and end times of transitions between indicated states based on a set of movies with varying lengths (movie durations in minutes: minimum, 85.2; maximum, 975; mean, 368; standard deviation, 210). (C) Probability distribution of zymosan state transitions color coded as above. The upper panel contains the distribution of transitions starting from surface bound zymosan (“S”). This probability was calculated over the entire course of time lapse observation. Of the zymosans that become fungipod associated, further transitions are possible as depicted in the lower panel showing state transitions starting from the fungipod associated zymosan (“F”) state. N = 301 total zymosan particles observed for a cumulative duration of ∼980 hours. (10.05 MB TIF) [file ppat.1000760.s003.tif]

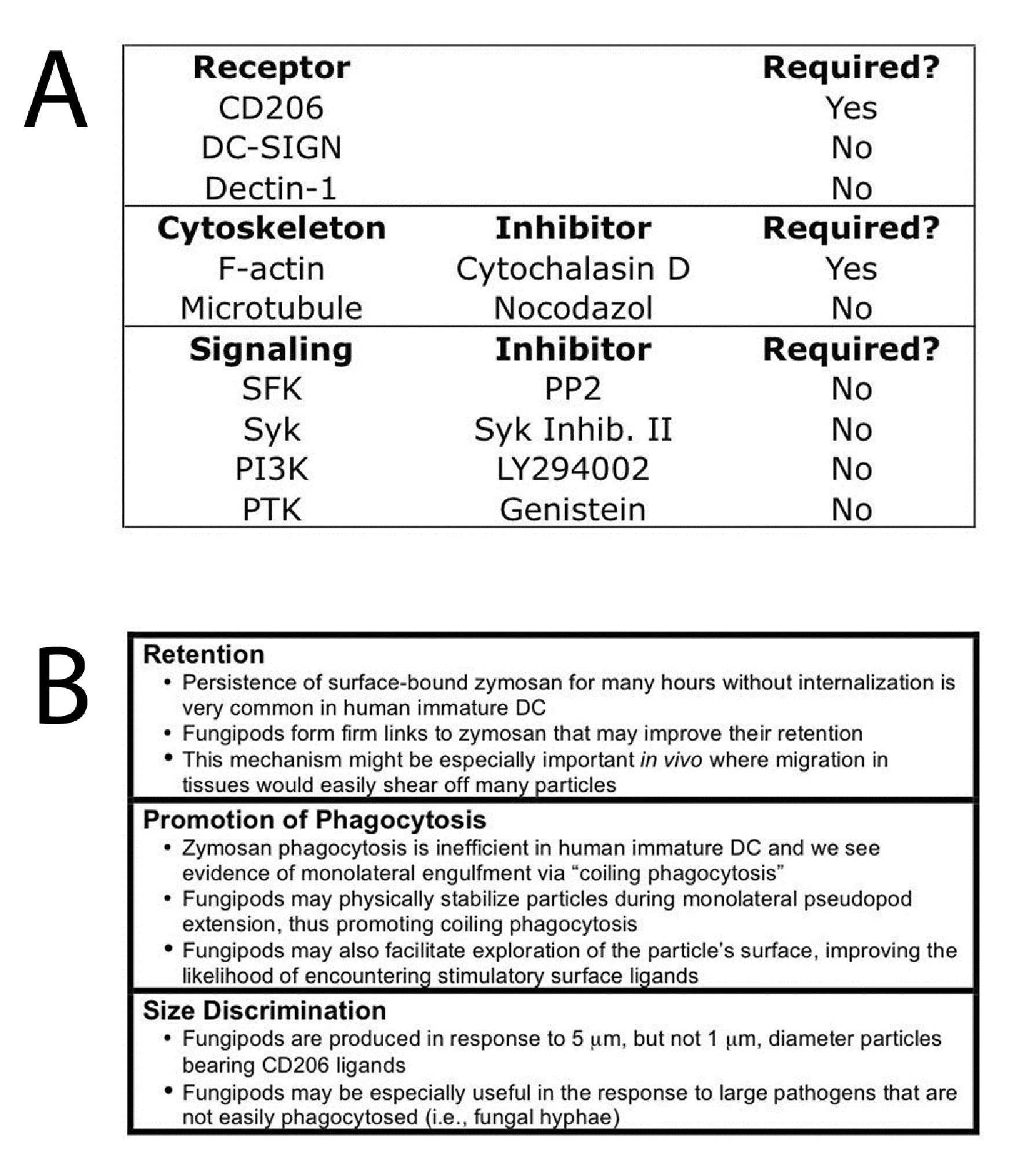

Supplement: Figure S4 — Tables summarizing our findings regarding the mechanism of fungipod formation (A) and perspectives on the functional significance of fungipods (B). Abbreviations: SFK, Src-family kinases; PI3K, phosphoinositide 3-kinase; PTK, protein tyrosine kinase. (3.58 MB TIF) [file ppat.1000760.s004.tif]
